# Withholding and canceling a response in ADHD adolescents

**DOI:** 10.1002/brb3.244

**Published:** 2014-06-26

**Authors:** Mehereen Bhaijiwala, Andre Chevrier, Russell Schachar

**Affiliations:** 1 Institute of Medical Science, University of Toronto7213 Medical Sciences Building, 1 King's College Circle, Toronto, Ontario, M5S1A8, Canada; 2 Neurosciences and Mental Health and the Department of Psychiatry, The Hospital for Sick Children555 University Avenue, Toronto, Ontario, M5G1X8, Canada

**Keywords:** ADHD, adolescents, fMRI, IFG, imaging, MPFC, proactive inhibition, reactive inhibition, response inhibition, stop-signal task

## Abstract

**Background:**

Deficient response inhibition in situations involving a trade-off between response execution and response stopping is a hallmark of attention deficit hyperactive disorder (ADHD). There are two key components of response inhibition; reactive inhibition where one attempts to *cancel* an ongoing response and prospective inhibition is when one *withholds* a response pending a signal to stop. Prospective inhibition comes into play prior to the presentation of the stop signal and reactive inhibition follows the presentation of a signal to stop a particular action. The aim of this study is to investigate the neural activity evoked by prospective and reactive inhibition in adolescents with and without ADHD.

**Methods:**

Twelve adolescents with ADHD and 12 age-matched healthy controls (age range 9–18) were imaged while performing the stop signal task (SST).

**Results:**

Reactive inhibition activated right inferior frontal gyrus (IFG) in both groups. ADHD subjects activated IFG bilaterally. In controls, prospective inhibition invoked preactivation of the same part of right IFG that activated during reactive inhibition. In ADHD subjects, prospective inhibition was associated with deactivation in this region. Controls also deactivated the medial prefrontal cortex (MPFC) during prospective inhibition, whereas ADHD subjects activated the same area.

**Discussion:**

This pattern of activity changes in the same structures, but in opposite directions, was also evident across all phases of the task in various task-specific areas like the superior and middle temporal gyrus and other frontal areas.

**Conclusion:**

Differences between ADHD and control participants in task-specific and default mode structures (IFG and MPFC) were evident during prospective, but not during reactive inhibition.

## Introduction

Response inhibition is a critical component of executive control. In general, there are two components to response inhibition. Reactive inhibition is involved when one tries to urgently *cancel* an ongoing action as a result of changing intentions, external circumstances or errors (Verbruggen and Logan 2008a). Reactive inhibition is akin to slamming on the breaks when a light suddenly turns red. Alternatively, one can *withhold* the initiation of an action until further information is available about whether the response should be executed or not and is referred to as prospective inhibition. Therefore, prospective inhibition can be viewed as being prepared to break at a stop sign at an upcoming intersection.

The interplay between prospective and reactive inhibition plays a crucial role in the control of movement (Band and van Boxtel 1999), in normal development (Harnishfeger and Pope 1996; Williams et al. 1999) and in the etiology of several psychiatric disorders such as attention deficit hyperactive disorder (ADHD) (Willcutt et al. 2005).

Reactive and prospective inhibition are both replicated deficits in ADHD (Barkley 1997; Schachar et al. 2000; Nigg et al. 2002; Lipszyc and Schachar 2010). Moreover, prospective inhibition is known to facilitate reactive inhibition; we stop faster when we know we *might need to*. However, the speed and efficiency of going and of stopping are believed to be independent in inhibitory control tasks (Band et al. 2003). People who go more slowly do not necessarily stop more efficiently than those who respond more quickly. Therefore, the interplay between prospective and reactive inhibition is not observable from behavioral data alone (Chevrier et al. 2007).

Separating the neural correlates of prospective inhibition from reactive inhibition would enhance our understanding of the nature of inhibitory control and the etiology of disorders marked by inhibitory control deficits. In this study, we use an established fMRI approach to separate neural activity during prospective inhibition from those during reactive inhibition in ADHD, a disorder characterized by poor inhibitory control (Schachar et al. 2005).

The stop signal task (SST) (Logan et al. 1984; Verbruggen and Logan 2008a) presents a laboratory analog of a real-life situation that can be used to investigate both prospective and reactive inhibition (Chikazoe et al. 2009; Verbruggen and Logan 2009). Trials in the SST consist of a warning signal followed by choice-response cue (respond with left hand to the letter X or right hand to O), here referred to as the “go task.” The “go phase” of the task refers to the period of time within a trial that follows the presentation of the go cue, but which precedes the appearance of any stop cues. The go phase contains neural activity associated with prospective inhibition that arises prior to presentation of the stop signal by definition. Occasionally (e.g., 33%), a stop signal follows the imperative stimulus, indicating that the subject should not respond on that trial (“stop task”). The “stop phase” of the task hence refers to this period of time that follows the presentation of a stop cue on successful stop trials. The stop phase contains neural activity associated with reactive inhibition. In the SST, delay between the presentation of the go stimulus and presentation of the stop signal (stop-signal delay) is dynamically adjusted so that responses can be successfully interrupted approximately half of the time. Responses on the other half of the trials are already too far underway to be canceled, and therefore constitute inhibition errors (Logan et al. 1984; Logan 1994).

The latency of the stopping process known as *stop-signal reaction time* (SSRT) is estimated by subtracting the mean stop-signal delay from the mean go reaction time (RT) (Logan et al. 1984, 1997) on trials that do not involve a stop signal (Logan et al. 1997). Shorter SSRT indicates better response inhibition (Logan et al. 1984; Band et al. 2003; Verbruggen and Logan 2008a). SSRT is a stable (Soreni et al. 2009) and heritable trait (Friedman and Miyake 2004; Schachar et al. 2005; Goos et al. 2007; Crosbie et al. 2013) that is highly associated with ADHD traits (Crosbie et al. 2013). Individuals with a diagnosis of ADHD have significantly *longer* SSRT than age-matched healthy controls. This is indicative of deficient response inhibition in patients with ADHD (Schachar et al. 2000) and also in individuals with a diagnosis of obsessive-compulsive disorder and schizophrenia (Lipszyc and Schachar 2010). Moreover, SSRT also appears to be a stable marker of genetic risk in ADHD (Bellgrove and Mattingley 2008; Crosbie et al. 2013).

The relative contributions of prospective and reactive inhibition, however, have not been studied in ADHD and may contribute to the understanding of the distinctive neurobiology of ADHD and other conditions (Bilder et al. 2009).

Previously, we developed a novel approach for imaging the sequence of prospective inhibition (which occurs on all trials in the SST) followed by reactive inhibition (which occurs only on successful stop trials). We isolated prospective inhibition from motor activities during the go phase of the task by contrasting right- and left-hand responses in order to preferentially conserve common cognitive activities while suppressing those specific to the handedness of the response. The resulting estimate of prospective inhibition activities that precede the appearance of stop signals could then be used to isolate activities underlying reactive inhibition that occurs after the presentation of stop signals. Using this approach in a group of healthy adults (Chevrier et al. 2007), we found that go-phase activity involved right prefrontal and midline regions, which we interpreted as being involved in the prospective inhibition of responses and monitoring for the potential need to stop, respectively. Reactive inhibition on successful stop trials activated the right IFG and basal ganglia (caudate) but did not activate midline regions implicated in prospective inhibition.

Several studies have investigated the role of prospective inhibition either by manipulating stop-signal probability (Vink et al. 2005; Chikazoe et al. 2009; Zandbelt and Vink 2010) or by examining the intertrial variability between groups (Hughes et al. 2013). In this study, we differentiate activity that occurs prior to the presentation of the stop signal from activity that follows the stop signal. Separating these phases of activity as done here can reveal group differences in prospective and reactive inhibition that otherwise would be masked as a result of averaging out the processes.

We apply this approach for the first time in adolescents with a diagnosis of ADHD and in age-matched healthy controls. We predict atypical prospective inhibition activity in ADHD based on existing evidence of difficulty withholding prepotent responses during neuropsychological tests (Firestone et al. 1998; Wright et al. 2014; in press), event related potential (ERP) studies which show delayed P300, an index of preparation (O'Connell et al. 2004; Liotti et al. 2010) and atypical default mode activity in ADHD during the transition from a resting state to a state of preparedness for task-related activity (Castellanos et al. 2006; Sonuga-Barke and Castellanos 2007). We also predict atypical reactive inhibition activity in the IFG based on considerable behavioral data (Lipszyc and Schachar 2010) and the results of previous fMRI studies (Rubia et al. 1999, 2003b, 2005; Schulz et al. 2004, 2005a,b; Smith et al. 2006).

## Method

### Participants

Data were acquired for 24 subjects (12 adolescents diagnosed with ADHD and 12 normal healthy control subjects) between the ages of 9–18 years. Participants gave informed, written consent and the Hospital for Sick Children institutional research ethics board approved the study. ADHD participants (*n* = 12) who had been taking stimulant medication were asked to stop 24 h prior to the scan in order to eliminate drug-induced BOLD changes (Dodds et al. 2008). Their past and present medication history was recorded as part of the diagnostic assessment protocol. Participants who were using medication other than stimulants were excluded from participating because certain medications like SSRI's and atomoxatine cannot be discontinued briefly for research. Current and previous use of stimulant medication was documented for evaluation of any possible treatment effect on performance or neural activity.

Participants and their parents were interviewed separately and together using the PICS-IV (Ickowicz et al. 2006). Intelligence was assessed using WISC-IV. In order to be included, ADHD subjects were required to meet the DSM –IV criteria for ADHD defined as having at least six of nine inattentive, six of nine hyperactive-impulsive symptoms or both according to at least two of three informants (parents, teacher, and patient self-report). ADHD subjects also had to show moderate to severe impairment at home and at school (Global Assessment Scale (Shaffer et al. 1983) score of less than 60). Participants were excluded if they had any comorbid psychiatric or neurological disorder other than oppositional defiant disorder or learning disability within the previous 12 months (e.g., obsessive compulsive disorder, Tourette syndrome, major depressive, anxiety or pervasive developmental disorder), an IQ score of below 80 on both verbal and performance scales or any medical problem that would impact fMRI participation. Subjects with metal braces or metal fragments in their body were also excluded due to contraindications in the MRI environment.

Nine ADHD participants were diagnosed with ADHD combined subtype and three met the criteria for the inattentive subtype. Two participants also met DSM-IV criteria for oppositional defiant disorder (ODD). Control subjects were assessed in a comparable manner and reported no psychiatric or medical disorders. All subjects were right-handed and had normal vision and hearing.

### The stop-signal task

The stop-signal task (SST) involves a primary choice reaction time task and a secondary stop task. Each trial began with a fixation point which appeared in the center of a black screen for 500 ms, followed by the go stimulus for 1000 ms. Participants were instructed to respond as quickly and as accurately as possible with their left thumb using a fMRI-compatible response box when the letter “X” appears on the screen or with their right thumb when the letter “O” appeared. In 33% of the trials, a stop signal (background color change from black to red) followed the go stimulus by some delay. Participants were told to stop their response if they saw the stop signal. They were told not to wait for stop signals. The initial stop-signal delay was 250 ms and was dynamically adjusted following each stop signal. When subjects successfully inhibited a response, the delay was increased by 50 ms on the next stop trial and when they failed to stop a response, the delay was decreased by 50 ms. Dynamic adjustment tracks to the delay and on average, individuals can stop 50% of responses when a stop signal is presented. Intertrial interval (ITI) was jittered such that trials were either 2.5 or 3.5 sec to ensure no multicollinearity of event types. The trials were jittered using random combinations of spread-spectrum binary coding sequences to maximize the number of independent equations in the deconvolution analysis, which enhances the separation of the event types in the experimental design. In order to establish a well-defined baseline of neural activity, every fourteenth trial was followed by a 17.5 sec rest period in which no stimuli were presented. This also ensured consistency with the bounds established by Ollinger et al. (2001) for the optimal separation of sequences in within-trial activities.

Trial order was pseudorandomized so that the current type of trial did not predict the subsequent kind of trial. The task involved 224 trials, requiring a total scan time of 15 min. The mean go response time (RT) was observable from the 67% of trials in which no stop signal appeared. The stop-signal reaction time (SSRT) was estimated by subtracting the mean delay on stop-signal trials from the mean go RT on no-signal trials.

### Analysis of behavioral data

All behavioral data were analyzed using SPSS (version 18, SPSS Inc., Chicago, Illinois). We assessed group differences in behavioral measures (SSRT, go reaction time, percentage of correct response (PCR), and percentage of successful inhibition (PSI). For adequate performance, the PSI was required to be approximately in the 50th percentile and PCR was required to be above 95 percent.

### Scanning parameters and data analysis

Imaging was done with a GE LX 1.5T MRI scanner (General Electric, Milwaukee). Anatomical data were acquired with a standard high-quality SPGR sequence (120 slices, 1.5-mm thick, FOV 24 cm, 256 × 256 matrix). Functional data were collected using a GRE-EPI sequence with a custom 8-channel head coil (TE = 40; TR = 2000; Flip angle = 90°; 24 slices; 6-mm thick; FOV 24 cm; 100-kHz readout bandwidth). These images were reconstructed to a 64 × 64 pixel resolution and final voxel size of 3.75 × 3.75 × 6 mm^3^. Behavioral data were collected using a Lumitouch fibre-optic button box (Lightwave Medical, Burnaby, BC, Canada) interfaced to a laptop running the stop task paradigm.

Functional data were analyzed using AFNI (Cox 1996). Images were motion corrected using a standard coregistration algorithm. Estimated motion parameters were inspected to ensure that the amount of absolute motion did not exceed 2 mm and angular displacement was not greater than 2°. We used a general linear model of stimulus vectors convolved with the hemodynamic response function (HRF) using AFNI's 3dDeconvolve program. Estimates of baseline and linear drift were generated along with 6-point HRF's (12-s duration, 4 sec delay) for each event type: fixate; X (left-hand response); O (right-hand response); stop (successful inhibition); and, error (failed inhibition).

Prospective inhibition activity common to both left- and right-hand responses was isolated using the contrast [½(X + O)]. This contrast suppresses activity specific to left- and right-hand responses, which only occur on a subset of trials trial, while enhancing neural activity that reflects *common* prospective inhibition, which is present in every trial of the SST. Activity associated with reactive inhibition on successful stop trials could then be estimated by removing the prospective inhibitory activity from successful stop trials using the contrast [stop–½(X + O)].

Intensity maps for the relevant contrasts (prospective inhibition = [½(X + O)] and reactive inhibition = [stop–½(X + O)]) were generated for individual subjects by taking the area under the HRF, warped into Talairach space, Gaussian blurred (6-mm FWHM), and resampled at 1 mm^3^ resolution. The single subject activation maps were passed on to a random effects ANOVA analysis that was conducted separately for the ADHD and healthy control groups in order to identify the general pattern of whole brain corrected activity for each group. Maps for ADHD and healthy controls were examined to identify qualitative differences in their patterns of activity.

Group difference maps were generated using a nested repeated-measures 3-factor ANOVA (participants, diagnostic status, and event types) in order to identify significantly different activities between healthy and ADHD adolescents. Group difference (Control-ADHD) for prospective and reactive inhibition activities from the ANOVA output were distributed as a t* statistic with 138° of freedom due to the number of regressors and subjects in the study.

Output from all the analyses (ADHD, control, and control-ADHD) were converted to raw *Z* scores and corrected for multiple comparisons using AFNI's AlphaSim program as in Chevrier et al. (2007). AlphaSim estimates the overall significance or the probability of a false detection (type 1 error) through a combination of voxel intensity thresholding and minimum cluster size thresholding, which enhances the power of the statistical test. AlphaSim can be considered a family-wise error rate procedure and provide a more stringent control over false discovery in comparison to false discovery rate (FDR) controls (Xiong et al.^1995^).This analysis required significant voxels to be part of a larger cluster of at least 6 original contiguous voxels (540 mm^3^) with a minimum *Z* score of 2.32, for an overall *α* of 0.046.

### Behavioral results

The ADHD and control groups differed marginally in age (*P* = 0.06) and Go RT (*P* = 0.08). There was no difference in IQ or in Stop Task performance as indexed by percentage correct response (PCR) (*P* = 0.42) and percentage of successful inhibition (PSI) (*P* = 0.38). Behavioral data confirmed that there were no differences in either the performance of the task or in the speed of the going process other than in the latency of the response inhibition process or SSRT (see Table 1). SSRT differed significantly between groups [t (22) = −2.217, *P* = 0.03] even after controlling for nonsignificant differences in age via hierarchical regression.

**Table 1:** Comparisons between controls and ADHD participants on age and other relevant psychometric indices

	ADHD (*N* = 12)		Controls (*N* = 12)		Sig.
		
	Mean	SD	Mean	SD	
Age	13.8	2.3	15.4	1.7	0.06
SSRT	238.0	53.3	198.1	34.8	0.03*
Go RT	636.2	145.2	542.2	105.7	0.08
PSI	51.9	3.7	50.8	2.3	0.38

SSRT, stop signal reaction time; Go RT, Go reaction time; PSI, probability of successful inhibition; PCR, percent correct response; SD, standard deviation.

Lists the means and standard deviations for the variables of interest. Significance is based on two-tailed *t*-test of significance.

* indicates statistical significance.

### Controls

During prospective inhibition, the control group exhibited positive activity in the right IFG (*x* = 31, *y* = 36, *z* = 10; *Z* score = 4.30, *P* < 0.00001, 747 mm^3^) and deactivation in the right medial prefrontal cortex (MPFC) (*x* = 1, *y* = 49, *z* = −3; *Z* score = −13.00, *P* < 0.00001, 7327 mm^3^). We also observed positive activation in the right middle frontal gyri (*x* = 33, *y* = 26, *z* = 20; *Z* score= 3.39, *P* = 0.0006, 745 mm^3^), left superior frontal gyrus (*x* = −19, *y* = 64, *z* = 19; *Z* score =12.99, *P* < 0.0001, 749 mm^3^), right superior temporal gyrus (*x* = 64, *y* = −45, *z* = 13; *Z* score = 3.76, *P* = 0.0002, 610 mm^3^), and inferior parietal lobe (IPL) (*x* = −42, *y* = −32, *z* = 54; *Z* score 3.50, *P* = 0.0005, 560 mm^3^). Deactivations were observed in the anterior temporal lobe (*x* = 51, *y* = 3, *z* = −3; *Z* score = −4.40, *P* < 0.0001, 4867 mm^3^), bilateral posterior insula (right: *x* = 37, *y* = −17, *z* = 3; *Z* score −3.48, *P* = 0.0005, 585 mm^3^; left: *x* = −43, *y* = 11, *z* = −4; *Z* score = −4.82, *P* < 0.0001, 2483 mm^3^), left supramarginal/post central gyrus (*x* = −52, *y* = −42, *z* = 33; *Z* score = −4.00, *P* < 0.00001, 965 mm^3^), left precuneus (*x* = −1, *y* = −52, *z* = 39; *Z* score = −3.51, *P* = 0.0004, 659 mm^3^), and various cerebellar areas.

During reactive inhibition, the control group elicited positive activity in the right inferior frontal gyrus (IFG) (*x* = 42, *y* = 20, *z* = 10; *Z* score = 3.06, *P* = 0.002, 636 mm^3^), in addition to positive activity in left IPL (*x* = −55, *y* = −48, *z* = 30, *Z* score = 4.04, *P* < 0.00001, 627 mm^3^), left fusiform (*x* = −31, *y* = −23, *z* = −11; *Z* score = 3.14, *P* = 0.001, 940 mm^3^), right posterior cingulate cortex (PCC) (*x* = 1, *y* = −81, *z* = 35; *Z* score = 5.89, *P* < 0.00001, 783 mm^3^), and left cerebellum (*x* = −31, *y* = −75, *z* = −49; *Z* score = 3.73, *P* = 0.0002, 958 mm^3^). They also showed a deactivation in the right cingulate gyrus (*x* = 5, *y* = 19, *z* = 40; *Z* score = −4.17, *P* < 0.0001, 888 mm^3^).

For a complete list of regions, please refer to Table S1, provided in the Supporting Information.

### Attention deficit hyperactive disorder

During prospective inhibition, the ADHD group exhibited deactivation in the right IFG (*x* = 37, *y* = 33, *z* = 13; *Z* score = −4.36, *P* < 0.00001, 653 mm^3^) as well as a deactivation in the right superior frontal (*x* = 20, *y* = 67, *z* = 10; *Z* scores = −4.09, *P* < 0.00001, 1008 mm^3^), left presupplementary area (*x* = −6, *y* = −5, *z* = 61; *Z* score = −4.71, *P* < 0.00001, 818 mm^3^), left superior temporal gyrus (*x* = −59, *y* = −30, *z* = 19; *Z* score= −4.54, *P* < 0.0001, 684 mm^3^), right middle temporal gyrus (*x* = 52, *y* = −46, *z* = 8; *Z* score = −4.47, *P* < 0.00001 569 mm^3^), posterior insula (right: *x* = 41, *y* = 23, *z* = 12; *Z* score 3.36, *P* < 0.00001, 1520 mm^3^; left: *x* = −36 *y* = −14, *z* = 16; *Z* score= −3.58, *P* = 0.0003, 624 mm^3^), PCC (*x* = 7, *y* = −57, *z* = 7; *Z* score = −4.31, *P* < 0.0001, 542 mm^3)^, and precuneus areas (*x* = 7, *y* = −81, *z* = 11; *Z* score = −4.88, *P* < 0.00001, 3015 mm^3^). ADHD subjects also showed positive activation in the inferior temporal gyrus (*x* = −43, *y* = −11, *z* = 33; *Z* score = 3.36, *P* < 0.0001, 624 mm^3^) and right cingulate gyrus (*x* = 20, *y* = 12, *z* = 25; *Z* score 3.18, *P* < 0.00001 1932 mm^3^).

During reactive inhibition, the ADHD group exhibited positive activity in the right IFG (*x* = 43, *y =* 12, *z* = 13, *Z* score = 3.36, *P* < 0.00001, 624 mm^3^), right superior frontal (*x* = 31, *y* = −54, *z* = 28; *Z* score = 4.71, *P* < 0.00001, 650 mm^3)^, right middle frontal (*x* = 46, *y* = 20, *z* = 39; *Z* score 4.24, *P* < 0.00001, 669 mm^3^), right (*x* = 9, *y* = 4, *z* = 15; *Z* score 3.87, *P* = 0.0001, 786 mm^3^) and left caudate (*x* = −6, *y* = −11, *z* = 13; *Z* score 3.36, *P* = 0.0007, 614 mm^3^), right IPL (*x* = 57, *y* = −41, *z* = 24; *Z* score 3.85, *P* = 0.0001, 669 mm^3^), and cuneus (*x* = 0, *y* = −78, *z* = 8; *Z* score 5.12, *P* < 0.00004, 1975 mm^3^) There were no significant negative activations in this map (Table 2).

**Table 2:** Activation foci during prospective and reactive inhibition in the ADHD group

Region	BA	Coordinates	*Z* score
Prospective inhibition			
Left medial frontal	6	−6, −5, 61	−4.71
Left Inferior frontal	45	−52, 13, 22	4.54
Right inferior frontal gyrus	46	54, 32, 10	−3.59
Right inferior frontal gyrus	46	37, 33, 13	−4.26
Right precentral gyrus	6	41, 8, −50	−3.82
Right superior frontal	10	20, 67, 10	−4.09
Left superior frontal	8	−19, 28, 54	−4.41
Right cingulate gyrus	24/32	20, 12, 25	3.18
Left posterior insula	13	−36, −14, 16	−3.58
Right posterior insula	13	41, 23, 12	−4.14
Left superior temporal gyrus	42	−59, −30, 19	−4.54
Right middle temporal gyrus	22	52, −46, 8	−4.47
Left inferior temporal gyrus	20	−43, −11, 33	3.36
Left parahippocampal gyrus		−5, 40, 6	−5.06
Left posterior cingulate	30	−6, −67, 10	−5.13
Right posterior cingulate	30	7, −57, 7	−4.31
Left precuneus	7	−22, −76, 45	−4.91
Right cuneus	17	7, −81, 11	−4.88
Left cuneus	18	−2, −97, 17	−5.03
Left cuneus	18	−12, −99, 10	−4.42
Right lingual	19	33, −57, 10	−3.64
Right middle occipital gyrus	39	−39, 74, 17	−4.58
Left middle occipital gyrus	39	−26, −89, 15	−4.51
Left lateral cerebellum		−1, −37, −35	−5.50
Reactive inhibition			
Right superior frontal gyrus	9/10	31, 54, 28	4.71
Right inferior frontal gyrus	44/13	43, 12, 13	3.36
Right middle/inferior frontal gyrus	9	48, 12, 30	3.73
Right middle frontal gyrus	8/9	46, 20, 39	4.24
Right caudate head		9, 4, 15	3.87
Left caudate head		−6, −11, 13	3.36
Right inferior parietal lobe	40	57, −41, 24	3.85
Right cuneus	19	7, −79, 37	5.17
Left cuneus	18	−0, −78, 8	5.12

### Neural activity differences between controls and ADHD participants

**Figure 1:**
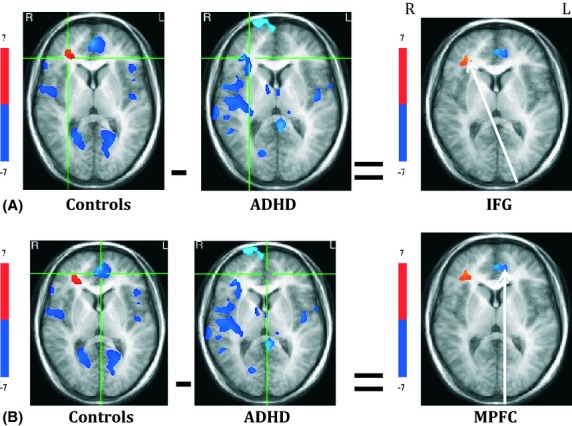
Differences between ADHD and control groups in the IFG and MPFC during prospective inhibition. Thresholded contrast map showing the differences between controls and ADHD (CTL-ADHD) in the IFG and MPF (Talairach z: 10). Red denotes activation while blue signifies deactivation. These contrasts indicate activation in the controls and deactivation in the ADHD group in the IFG and deactivation in the MPFC in controls and activation in the ADHD group. (A) Denote activity differences in the IFG. (B) Show activity differences in the MPFC. All images have been corrected for an overall *α* < 0.05. R/L: right/left.

Consistent with our prediction, a significant difference in activity between ADHD and control groups during the go phase was present in right IFG as a result of normal controls *pre*-activating this region prior to the onset of stop cues, whereas ADHD subjects were *de*-activating this region (Fig. 1). Also consistent with our prediction, a significant difference between ADHD and controls during prospective withholding was evident in the medial prefrontal frontal cortex (MPFC), an area associated with default mode function (Fig. 1). Normal control subjects *de*-activated the MPFC, whereas ADHD subjects *pre*-activated this region. Therefore, differences in IFG and MPFC between ADHD and controls were not a result of over or under-activation of a region, but rather as a result of activation in one group and negative activation in the other. We observed the same pattern for every other significant difference during prospective inhibition. Significant differences were either the result of controls activating and ADHD subjects de-activating a given region as seen in the right IFG, superior and middle temporal lobes, right IPL and anterior insula or controls de-activating and ADHD subjects activating as observed in the right MPFC and left post central gyrus (see Table 3).

**Table 3:** Between-group differences during prospective and reactive inhibition

Area	BA	Coordinates	Group intensity	Control intensity	ADHD intensity
Prospective inhibition					
Right medial prefrontal cortex	32	2, 47, −3	−22.2	−20.53	1.70
Right inferior frontal gyrus	47	33, 36, 6	7.80	3.39	−6.91
Left postcentral gyrus	3	−42, −20, 58	4.40	−6.12	−2.68
Right inferior parietal lobe	40	−30, −36, 40	4.57	2.52	–2.38
Right superior temporal lobe	22	49, −18, −8	8.07	2.50	−5.57
Right Middle temporal lobe	39	−54, −59, 26	5.28	2.44	−2.84
Left middle temporal lobe	39	−42, −49, 14	6.50	2.90	−3.60
Left insula	13	−36, 6, 17	6.38	1.08	−5.24
Right insula	13	44, −23, 15	9.02	1.07	−7.93
Reactive inhibition					
Right medial frontal gyrus	8	3, 45, 39	−9.68	−4.51	5.17
Right middle frontal gyrus	6	26, −2, 52	4.17	3.68	−0.49
Right superior frontal gyrus	10	22, 66, 18	−10.57	−2.39	8.18
Right middle temporal gyrus	21	−53, −9, −12	6.55	2.53	−4.02
Left cerebellum		−7, −68, −28	6.19	4.01	−2.18

Contrary to our predictions, the ADHD and control groups did not differ in IFG activity during reactive inhibition; both groups activated this region to a similar extent when stop signals actually appeared and subjects successfully stopped their response. However, significant differences were present in several other regions. As with prospective inhibition, group differences during reactive inhibition were also primarily the result of opposite departures from baseline activity. Significant differences in the right superior and middle frontal, middle temporal, and left cerebellum during reactive inhibition were either the result of activation in healthy control subjects and negative activation in ADHD subjects or negative activation in healthy control subjects and activation in ADHD subjects (see Table 3). We also conducted additional Pearson correlation analysis to assess the relationship between SSRT and activation in the right IFG and there were no significant associations to report in either group.

**Figure 2:**
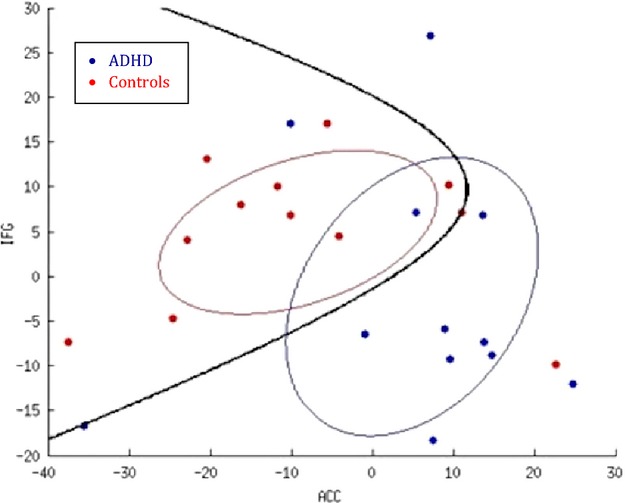
Quadratic Discriminant Analysis (QDA) using IFG and MPFC activity in the ADHD and Control Participants. QDA revealed that the ADHD group congregated to the bottom quadrant (with negative activity in the IFG and positive in the MPFC) and the control group were mostly located in the top quadrant (positive activity in the IFG and negative in the MPFC) during prospective withholding. Each point signifies the activity in the IFG and MPFC for a single subject. ADHD subjects are in blue and Controls are in red.

Medial prefrontal cortex and IFG activities in normal controls and ADHD groups during prospective withholding are plotted in Figure 2 as proxies for task-specific and default mode network activity (Fig. 2). Healthy control subjects were clustered in the upper left quadrant, associated with activation of the task-specific IFG, and *de*-activation of default mode MPFC. By contrast, ADHD subjects, while less clustered than healthy control subjects, were generally distributed in the bottom right quadrant of this plot, associated with *de*-activation of the task-specific IFG and activation of default mode-related ACC. Post hoc quadratic discriminant functional analysis (DFA) on this plot (in SPSS) was capable of classifying subjects based on their prospective withholding activity in these two regions alone. The discriminant analysis assigns a posterior probability of being in a particular group. A loading factor for the variates above 0.30 is generally considered to be a meaningful contributor to the discriminant score. In our study, the two discriminants, that is, IFG and MPFC activities had loadings of -0.59 and 0.76, respectively.

## Discussion

We used the stop-signal task to separate neural activity related to prospective inhibition of a response from activity associated with reactive inhibition in adolescents with and without ADHD. The approach provided an estimate of prospective inhibition-related activity during the go phase of the stop task by explicitly accounting for the handedness of responses in the deconvolution analysis and by using a statistical contrast that enhanced cognitive activities common to left- and right-hand responses while suppressing activities specific to the lateralized motor response. This contrast also provides an optimal baseline from which to estimate activity attributable to reactive inhibition. Stop trials involve prospective inhibition activity that is similar to that on go trials, but do not actually contain an overt motor response. Therefore, estimating go-phase activity on stop trials using a contrast that suppresses hand-specific response-related activity is preferable to using a single regressor for all go trials because common and hand-specific go-phase activities are not statistically independent and should be accounted for in a simultaneous regression analysis. Therefore, both theoretical and empirical evidence support the use of the current method for separating prospective inhibition from reactive inhibition (Chevrier et al. 2007).

Previous analytical approaches were not designed to distinguish prospective from reactive forms of inhibition within the same task. More specifically, subtracting go trial activity from stop trial activity is a method that is routinely employed to isolate inhibition (Paloyelis et al. 2007; Li et al. 2008). This approach is based on the assumption that inhibition-related activity is evident only during the stop phase and that subtraction of go-phase activity from stop activity would isolate neural activity involved in “stopping” a response. However, these approaches do not separate the prospective inhibition that precedes stop signals from response-related activity. Alternatively, failed inhibition has been subtracted from successful inhibition in order to capture the neural substrates of inhibition while controlling for stop stimuli that are common to both failed and successful stop trials, but may in fact be identifying regions that are more associated with error detection than processing the sensory properties of the stop cues (Li et al. 2008).

We observed right IFG activity during reactive inhibition in healthy controls replicating the results of previous studies of inhibition in healthy adolescents and in adults (Rubia et al. 2003a; Aron et al. 2004, 2007; Aron and Poldrack 2006; Chevrier et al. 2007). We *also* observed IFG activation and MPFC negative activation in healthy controls during inhibition. IFG activation prior to the appearance of the stop signal has been reported previously by Chikazoe et al. (2009) and indicates that prospective inhibition activates task-specific areas of IFG pending information about whether the current trial will be a go or a stop trial. (Cf. please refer to Vink et al. 2005; Zanbelt and Vink 2010, for the role of the striatum in response inhibition).

Inferior frontal gyrus activation in prospective inhibition indicates a broader role for IFG as suggested by Hampshire et al. (2010). The right IFG in particular has been implicated in the selection of task-specific information, sustained attention and vigilance (Fassbender et al. 2004; Fassbender and Schweitzer 2006; Shallice et al. 2008) and suppression of noncritical information (Duncan and Owen 2000; Cole and Schneider 2007). It appears that IFG activity during prospective inhibition *c*ould reflect a readiness to respond to changing conditions by shifting attention to sudden salient events during the stop phases (e.g., reacting to the sudden appearance of a stop signal while a motor response is already underway) (Corbetta and Shulman 2002; Downar et al. 2002; Duann et al. 2009). The right IFG may, therefore, be instrumental in prospective inhibition via its role in attentional control (Duncan and Owen 2000), which is operationalized as an ability to rapidly adapt to changing salient stimuli (Corbetta and Shulman 2002; Hampshire et al. 2010; Sharp et al. 2010).

Participants with ADHD had significantly longer SSRT, which indicated deficient inhibitory control (Verbruggen and Logan 2008b) when compared to controls. They also did not differ in the latency of their go responses that suggested that their longer SSRT were not related to longer latencies of the go response (Logan et al. 1984). Additionally, there was no association between SSRT scores and neural activity in the IFG. However, the lack of association between two variables does not preclude the possibility of a functional dependence between the IFG and impaired inhibition process.

Attention deficit hyperactive disorder participants showed *negative activation* in the IFG and *activation* in the MPFC during inhibition but comparable activity to controls in IFG during inhibition. This pattern is in contrast to previous studies that consistently report negative activation in the IFG during inhibition (Pliszka et al. 2000; Durston et al. 2003, 2004, 2011; Booth et al. 2005; Rubia et al. 2005, 2008, 2009, 2011; Schulz et al. 2005b; Cubillo et al. 2010; Dibbets et al. 2010; Passarotti et al. 2010). Prior studies did not differentiate between the reactive and prospective phases of response inhibition. Consequently, their results combined negative activity during prospective inhibition and positive activity during reactive inhibition, which looked like a simple hypoactivation when compared to controls (Chevrier et al. 2007).

Negative activation in the MPFC in control groups during prospective inhibition supports the role of the ACC in default mode network processing (Gusnard et al. 2001b; Raichle et al. 2001; Greicius et al. 2007; Raichle and Snyder 2007). Failure to suppress the default mode network while performing the stop task appears to be associated with poor stopping ability (Congdon et al. 2010) and has previously been observed in an ADHD population (Sonuga-Barke and Castellanos 2007; Fassbender et al. 2009).

Studies of default mode network support the hypothesis of atypical preparation in ADHD. The default mode network is associated with spontaneous attentional fluctuations (Sonuga-Barke and Castellanos 2007) and is involved in monitoring internal states in resting conditions (Gusnard et al. 2001b). It is believed that crucial nodes in the default network such as MPFC disengage at moments when we need to react to changing external conditions (Raichle et al. 2001). Failure to suppress the default mode can interfere with the ability of task-specific networks to effectively regulate goal-directed behavior (Gusnard et al. 2001a; Raichle et al. 2001; Fair et al. 2007) such as the ability to stop a response (Congdon et al. 2010). Moreover, the magnitude of negative activation in the MPFC appears to be associated with greater attention and preparation in healthy adolescents when compared to individuals with ADHD (Fassbender et al. 2009).

Consistent with previous findings, our results indicate a failure to transition from the default mode to task-oriented networks in the ADHD group which in turn may affect their ability to inhibit a motor response. In fact, our results not only show a failure to properly *disengage* default mode networks and *engage* task-related networks during prospective inhibition, but further demonstrate that the ADHD subjects are in fact doing the *opposite* during prospective inhibition: namely, *disengaging* task-related circuits while *engaging* default mode circuits more intensely.

This interpretation is supported by the observation that in our sample, the ADHD group showed negative activity and controls showed positive activity in areas that are critical for efficient task completion. For example, during the go phase, the control group exhibited positive activity and the ADHD group displayed negative activity in the superior and middle temporal regions and anterior insula. Superior and middle temporal regions are involved in mental preparation (Kounios et al. 2006; Tian et al. 2011) recalling semantic rules necessary for task completion (Simmons and Martin 2009; Simmons et al. 2010). Positive activity in the temporal lobes is also associated with lower intraindividual variability in reaction time (Spinelli et al. 2011); which is believed to be a marker for preparation and alertness (Fassbender et al. 2009). The insula, in conjunction with temporal areas are critical nodes in the salience network (Seeley et al. 2007) that mediates a switch to relevant stimuli and recruits task appropriate regions. Deactivation in the ADHD group and positive activity in the controls were also evident in multiple frontal areas and the IPL that are critical for vigilance and ensuring flexibility in the decision-making process (Stuss and Alexander 2000).

In short, the pattern of preparatory activity in the right structures, but in the wrong direction compared to baseline, suggests that the inhibitory control deficit in ADHD could be associated with inappropriate tuning of networks involved in preparedness and attention to the task at hand.

These activity differences between ADHD and controls, consistently being the result of activity changes in opposite directions with respect to baseline, can perhaps be explained through the dysfunctional dopamine modulation in ADHD. Several studies have identified abnormalities in dopaminergic neurotransmission systems in an ADHD population (Rowe et al. 1998; Waldman et al. 1998; Swanson et al. 2000; Sonuga-Barke 2003; Sagvolden et al. 2005; Tripp and Wickens 2008; Bellgrove et al. 2009). Therefore, there is a reasonable possibility that abnormal dopaminergic functioning may be an important factor contributing to the inhibitory impairments evident in ADHD.

## Conclusion

This is the first study to differentiate neural activity during reactive inhibition and prospective inhibition in ADHD and normal control adolescents. We adopted a methodology that makes it possible to investigate these cognitive processes during inhibition within the confines of a single task.

It should be noted that while the current approach separates prospective from reactive inhibition, it is currently not possible to distinguish between prospective inhibition, monitoring, and the wave of various simultaneous cognitive processes and central response activations that precede the lateralization of the motor response that also resides in the go phase. We chose to focus on one aspect of the cognitive activity that lives in the go phase and future advancements in analytical techniques may make it possible to extricate the influence of events that occur almost simultaneously in time.

The next step in our research is to investigate the reinforcement-learning signals that arise following errors in an ADHD sample. The aim is to provide a clearer view of how processes like error detection and the subsequent modification after failed stop trials affect inhibitory control in normal and ADHD groups and the role of dopamine-regulated activities in models of stop-signal task performance in health and disease.

## Supporting Information

**Table S1.** List of activations during prospective and reactive inhibition in the Control Group. **Figure S1.** Whole-brain activity in the IFG and MPFC in controls and ADHD.
