# Supplementary material for: Withholding and canceling a response in ADHD adolescents
Source: Brain Behav. 2014 Jun 26;4(5):602–14. doi: 10.1002/brb3.244 (PMC4086366; doi:10.1002/brb3.244)
Supplement: Supplementary file 1 [file brb30004-0602-SD1.docx]

| - **Area** | - **BA** | - **Coordinates** | - **Z score** |
| --- | --- | --- | --- |
| - **PROSPECTIVE INHIBITION** |  |  |  |
| - Right anterior cingulate | 24/32 | - 1 49 -3 | - -13.00 |
| - Left superior frontal gyrus | - 10 | -19 64 19 | - 12.99 |
| - Right middle frontal gyrus | - 8/9 | - 33 26 20 | - 3.39 |
| - Right middle frontal | - 10/11 | - 34 44 -4 | - 5.07 |
| - Right middle frontal gyrus | - 9 | - 28 20 29 | - 3.34 |
| - Right inferior frontal gyrus | - 46 | - 31 36 10 | - 4.30 |
| - Left inferior frontal gyrus | - 47 | - -43 22 1 | -4.35 |
| - Left precentral | - 4/3 | - -26 -26 68 | - 4.77 |
| - Left precentral | - 4 | - -55 -5 26 | - -3.38 |
| - Right ventral posterior thalamic nucleus | - 27 | - 17-18 0 | - -2.91 |
| - Left thalamic medial geniculum body | - 27 | - -17 -28 -2 | - -4.70 |
| - Left inferior parietal lobe | - 40 | - -46 -32 54 | - 3.50 |
| - Left supramarginal | - 40 | - -52 -42 33 | - -4.00 |
| - Right posterior insula | - 13 | - 37 -17 3 | - -3.48 |
| - Left posterior insula | - 13 | - -43 11 -4 | - -4.82 |
| - Anterior temporal lobe | - 38 | - 51 3 -3 | - -4.40 |
| - Right superior temporal gyrus | - 22 | - 64 -45 13 | - 3.76 |
| - Left superior temporal gyrus | - 22 | - -47 -54 19 | - 3.97 |
| - Left caudate tail |  | - -20 -44 15 | - 3.66 |
| - Left precuneus | - 7 | - -1 -52 39 | - -3.51 |
| - Left precuneus | - 19 | - -9 -83 40 | - -4.73 |
| Left Tonsil (medial) |  | - -4 -47 -41 | - -5.25 |
| - Left inferior |  | - -20 -72 -37 | - -4.75 |
| - **REACTIVE INHIBITION** |  |  |  |
| Right inferior frontal gyrus | 13/44 | 42 20 10 | 3.06 |
| Right inferior frontal gyrus | 47 | 49 12 0 | 4.06 |
| Right cingulate | 32 | 5 19 40 | -4.17 |
| Left inferior parietal lobe | 40 | 55 -48 30 | 4.04 |
| Right supramarginal gyrus |  | 30 -22 -10 | 3.09 |
| Left fusiform gyrus | 37 | -39 -43 -9 | 2.69 |
| Left hippocampus | 36 | -31 -23 -11 | 3.14 |
| Right cuneus/posterior cingulate | 19 | 1 -81 35 | 5.89 |
| Cerebellar areas |  |  |  |

Table I: List of activations during prospective and reactive inhibition in the Control Group.

**Supplementary figure 1: Whole brain activity in the IFG and MPFC in controls and ADHD.**

Right Left

**
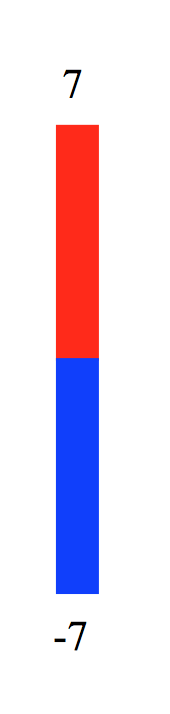

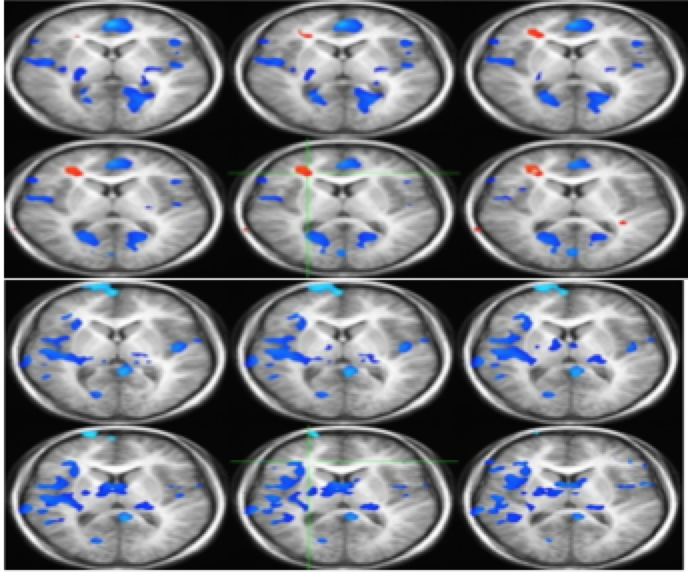
**

Controls

ADHD

Figure I: shows whole brain activation in the IFG and MPFC in Controls and ADHD. Red denotes activation while blue signifies deactivation. These contrasts indicate activation in the Controls and deactivation in the ADHD in the IFG and deactivation in the MPFC in controls and activation in the ADHD group. All images have been corrected for an overall α <0.05.
